# Lymphogranuloma Venereum Mimicking Locally Metastatic Rectal Cancer in an HIV-Negative Man

**DOI:** 10.7759/cureus.20216

**Published:** 2021-12-06

**Authors:** Sara Soliman, Pia Dogbey, Samuel Pan

**Affiliations:** 1 Internal Medicine, Waterbury Hospital, Waterbury, USA; 2 Infectious Diseases, Waterbury Hospital, Waterbury, USA

**Keywords:** lymphogranuloma venereum, hiv, rectal cancer, proctitis, msm

## Abstract

Lymphogranuloma venereum (LGV) can present as a sexually transmitted anorectal syndrome and is caused by *Chlamydia trachomatis* serotypes L1, L2, and L3. It was rare in the western world until a recent outbreak among men who have sex with men (MSM) in Europe and North America. Limited availability of diagnostic tests differentiating LGV from non-LGV *C. trachomatis* can make the diagnosis challenging.

We present a 33-year-old MSM with high-risk sexual behavior and anal atypical squamous cells of undetermined significance (ASCUS), who was evaluated for rectal pain, bleeding, constipation, and weight loss. Computed tomography of the abdomen and pelvis showed rectal wall thickening with pelvic adenopathy, concerning rectal carcinoma, also seen on colonoscopy as a 50% circumferential ulcerating rectal mass. The rectal swab was positive for *C. trachomatis* by immunofluorescence assay. Pathology confirmed severe active proctitis, but no malignancy. He was treated for presumed LGV proctitis with marked improvement. The case highlights an unusual presentation of LGV with severe inflammation and mass formation mimicking rectal carcinoma. Early identification of possible LGV especially in high-risk patients allows early appropriate treatment.

## Introduction

Lymphogranuloma venereum (LGV) is one of the less common sexually transmitted ulcerating anogenital diseases, which was previously perceived to be only endemic in parts of Africa and Asia until multiple outbreaks were described in Europe and the United States in 2003 [1-3]. More recent outbreaks have been reported among men who have sex with men (MSM) in Europe and North America [3-5], with a high prevalence of HIV co-infection [6,7]. It is caused by *Chlamydia trachomatis* serotypes L1, L2, and L3, manifesting initially as a transient genital ulcer, then a secondary inguinal lymph node infection and anorectal syndrome, and sometimes a late-stage anorectal fibrosing disease [1].

Due to potentially overlapping clinical presentations and radiological findings with non-LGV *C. trachomatis* infections or rectal malignancy [8,9], and the limited availability of diagnostic tests that differentiate LGV from non-LGV *C. trachomatis* infections, test results may be delayed, and appropriate and timely diagnosis can be challenging. History taking is particularly important to identify high-risk patients.

LGV should be considered in the differential diagnosis of high-risk patients presenting with proctitis or an ulcerating rectal mass, tested for chlamydia infection, and treated empirically once. This case aims to raise the awareness of LGV as an emerging sexually transmitted disease among the MSM community in the United States, even in those who are HIV-negative. Early identification and prompt management can reduce complications and limit the spread of infection.

## Case presentation

A 33-year-old MSM presented with eight days of severe rectal pain, intermittent blood per rectum, nausea, non-bloody vomiting, tenesmus, and constipation. He had decreased oral intake, epigastric and crampy lower abdominal pain, generalized fatigue, subjective fever, and chills. No inguinal swelling, genital ulcers, or penile discharge were noted.

He engaged in unprotected receptive anal and oral intercourse, and the last sexual encounter was two months prior to presentation. Other medical history included a positive anal Pap smear with 14 high-risk human papillomavirus (HPV) serotypes and atypical squamous cells of undetermined significance (ASCUS) on cytology, depression, and post-traumatic stress disorder from sexual abuse as a child. He had a history of rectal chlamydia at the age of 20 years.

Outpatient medications were clonidine, paroxetine, and emtricitabine-tenofovir disoproxil for pre-exposure prophylaxis (PrEP). There was no family history of malignancies.

The initial temperature was 99.6°F, blood pressure was 159/63 mmHg, and pulse rate was 53 beats/minute with normal oxygen saturation on room air. Physical exam showed a tender epigastrium and lower abdomen with guarding, but no rebound tenderness and no inguinal adenopathy. His oropharynx had mild erythema without any exudates, ulcers, or thrush. The rectal exam was limited due to discomfort, but no palpable masses were found.

White cell count was 11.9 x 109/L, hemoglobin level was 14.2 g/dl, and potassium level was 3.4 mmol/L, with otherwise normal renal and liver function. Urinalysis showed traces of protein and mild ketonuria attributed to starvation from poor oral intake.

**Figure 1:**
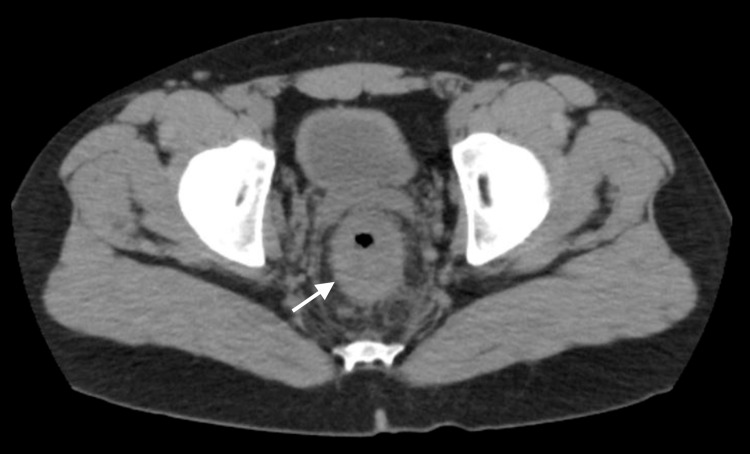
CT colonography with contrast shows significant thickening of the rectal wall, suggestive of rectal carcinoma.

CT scan of the abdomen and pelvis with contrast showed a markedly abnormally thickened rectal wall with multiple enlarged perirectal and inferior mesenteric lymph nodes, concerning rectal carcinoma with metastatic spread to lymph nodes (Figure 1).

**Figure 2:**
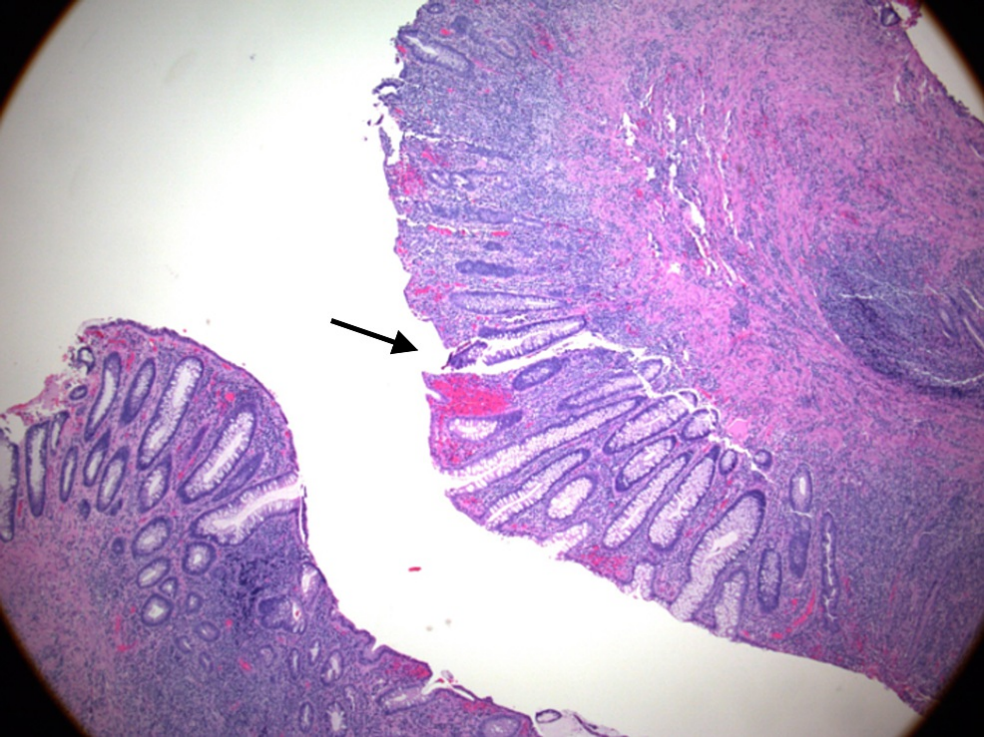
Rectal mass biopsy shows ulceration and reactive epithelial changes associated with inflammation with no evidence for dysplasia or malignancy.

A colonoscopy was performed by colorectal surgery and revealed a low ulcerated 50% circumferential rectal mass just above the dentate line extending to 7 cm. Rectal biopsy showed chronic proctitis with severe activity and erosion but no evidence for dysplasia or malignancy (Figure 2).

Despite the fact that there was no histological evidence of neoplasia, the abrupt transition of rectal mucosa from mass-like thickening to normal mucosa with associated lymphadenopathy remained concerning, leading to a second biopsy by proctoscopy, which again showed reactive epithelial changes associated with severe inflammation and ulceration and no evidence of neoplasia. Although the imaging findings were concerning for a rectal malignancy, the epidemiological and clinical presentation would also be strongly suggestive of proctitis due to a sexually transmitted disease (STD).

HIV 1 and 2 antigen/antibody (Ag/Ab) test by fourth-generation assay was negative, HIV RNA polymerase chain reaction (PCR) was <20copies/mL (not detected), rapid plasma regain (RPR) was non-reactive, and hepatitis B, C, and A viral screens were also negative. He was treated empirically with intramuscular ceftriaxone 250 mg once, and azithromycin 1,000 mg orally once for gonococcal and chlamydia proctitis.

The most likely diagnosis was chlamydia proctitis, specifically LGV, given the presence of a rectal mass on CT imaging, as LGV serotypes tend to invade lymphatics with resultant inflammation and possible fibrosis [2]. Confirmation became challenging as our hospital laboratory did not have the capability to differentiate LGV from non-LGV chlamydia serotypes by nucleic acid amplification test (NAAT). In consultation with an infectious disease specialist, empiric doxycycline 100 mg twice daily for LGV was started pending testing.

Rectal swab for gonorrhea and chlamydia by NAAT transcription-mediated amplification (TMA) (APTIMA® COMBO 2 Assay, Gen Probe®, San Diego, CA) was positive for both gonorrhea and chlamydia. Oropharyngeal swabs and urine NAAT for both chlamydia and gonorrhea were negative. *Chlamydia trachomatis* RNA immunofluorescence assay (IFA) revealed high titers for immunoglobulin G (1:1024) and immunoglobulin A (1:128), but negative immunoglobulin M (<1:10).

The final diagnosis was second-stage recurrent LGV proctitis with rectal mass. We found he had previously been prescribed oral doxycycline 100 mg twice daily for 21 days for a positive rectal chlamydia NAAT prior to being prescribed PrEP, three months prior to admission. He was discharged home after seven days once he tolerated oral food with a plan to notify all sexual partners within the prior 60 days to get tested and treated. His symptoms completely resolved on follow-up after completing a 21-day course of doxycycline.

## Discussion

LGV is an STD that became rare in developed countries since the introduction of antibiotics. It was more endemic in parts of Africa, Asia, South and Central America, and the Caribbean until 2003 when outbreaks were increasingly reported in countries in Europe, North America, and Australia, primarily in MSM [1,3,4]. The exact prevalence in the United States is unknown, due to the limited availability of LGV serovars-specific molecular tests, and the deletion of LGV from the national public health surveillance system in 1994. The majority of LGV cases are thus treated empirically and left unreported [2]. Previous studies have shown a high co-infection with HIV among MSM with LGV, ranging from 67% to 100% with a pooled prevalence estimate of 77.9% (95% CI 75.0-80.8%) in a systematic review of 13 studies between 2000 and 2009 [6]. HIV has been shown to be an independent risk factor for anorectal LGV infection, and other STDs may also be present including gonorrhea, syphilis, and genital herpes, which may all cause proctitis [7]. Our patient was HIV negative but had rectal gonorrhea, highlighting the need for additional STD testing, but also to consider LGV in the appropriate clinical setting in MSM regardless of HIV status.

Three clinical stages of LGV are described including a self-limiting primary stage with a painless genital papule or ulcer at the site of inoculation, followed weeks to months later by a secondary stage characterized by inguinal lymphadenopathy (buboes) and proctitis. If untreated, a tertiary stage with anogenital fibrosis resulting in strictures and fistula formation may follow [1,2,10]. Genital tract and inguinal lymph node infections have become less common. Proctitis or proctocolitis manifesting with rectal pain, tenesmus, mucoid discharge, hematochezia, abdominal pain, and constipation, with or without systemic signs of infection, are now the more common presentation, especially amongst MSM [1,10], as was the presentation in our patient. Early case recognition and treatment will prevent the spread of infection and progression to fibrosis and other complications.

Radiological findings of LGV can be confused with rectal malignancy, and include bowel edema, circumferential rectal wall thickening with associated fat stranding, and pelvic or retroperitoneal lymphadenopathy and other cases have been reported in the literature [8,9].

Diagnosis of LGV requires two steps, i.e., *C. trachomatis* NAAT followed by LGV biovar-specific DNA NAAT from the same sample used in the first step test [2]; however, diagnosis has been difficult because laboratory tests are not well standardized in the USA. Bacterial culture lacks sensitivity for rectal chlamydia infection compared to NAAT (36.1-47% versus 100%) [11]. Urethral specimens are usually negative in anorectal infections [2], and interpretation of chlamydia serologies is not yet standardized. NAATs were not FDA approved for extra-genital specimens until May 23, 2019; further, PCR-based genotyping to identify L1-L3 serovars to differentiate LGV from non-LGV *C. trachomatis* is not widely available, which can lead to diagnostic delays or missed diagnoses.

## Conclusions

LGV should be considered as a differential in MSM presenting with proctitis or rectal mass, even without HIV co-infection, and even in the absence of the well-known enlarged inguinal lymph nodes (buboes). In the situation where LGV serovar-specific genotype testing is unavailable, a presumptive diagnosis based on epidemiology, clinical features, and rectal NAAT for chlamydia should be made, and empiric therapy with oral doxycycline 100 mg twice per day for 21 days promptly initiated. Other STDs, including HIV, must be tested for as co-infection is common, and partner notification must be a part of the comprehensive treatment approach.
